# Extrinsic Calibration of Camera Networks Using a Sphere

**DOI:** 10.3390/s150818985

**Published:** 2015-08-04

**Authors:** Junzhi Guan, Francis Deboeverie, Maarten Slembrouck, Dirk van Haerenborgh, Dimitri van Cauwelaert, Peter Veelaert, Wilfried Philips

**Affiliations:** Image Processing and Interpretation, TELIN, Ghent University/iMinds, Sint Pietersnieuwstraat 41, 9000 Gent, Belgium; E-Mails: Francis.Deboeverie@telin.ugent.be (F.D.); Maarten.Slembrouck@telin.ugent.be (M.S.); dirk.vanhaerenborgh@telin.ugent.be (D.H.); Dimitri.VanCauwelaert@telin.ugent.be (D.C.); Peter.Veelaert@ugent.be (P.V.); philips@telin.UGent.be (W.P.)

**Keywords:** Camera network, extrinsic calibration, sphere-based calibration, orthogonal procrustes

## Abstract

In this paper, we propose a novel extrinsic calibration method for camera networks using a sphere as the calibration object. First of all, we propose an easy and accurate method to estimate the 3D positions of the sphere center w.r.t. the local camera coordinate system. Then, we propose to use orthogonal procrustes analysis to pairwise estimate the initial camera relative extrinsic parameters based on the aforementioned estimation of 3D positions. Finally, an optimization routine is applied to jointly refine the extrinsic parameters for all cameras. Compared to existing sphere-based 3D position estimators which need to trace and analyse the outline of the sphere projection in the image, the proposed method requires only very simple image processing: estimating the area and the center of mass of the sphere projection. Our results demonstrate that we can get a more accurate estimate of the extrinsic parameters compared to other sphere-based methods. While existing state-of-the-art calibration methods use point like features and epipolar geometry, the proposed method uses the sphere-based 3D position estimate. This results in simpler computations and a more flexible and accurate calibration method. Experimental results show that the proposed approach is accurate, robust, flexible and easy to use.

## 1. Introduction

Camera networks with common views have many applications in computer vision such as people tracking and 3D reconstruction. Extrinsic and intrinsic calibration is essential in these applications to ensure the geometric accuracy of 3D scene analysis. The intrinsic calibration parameters define the imaging geometry and the optical characteristics of each camera individually. The extrinsic parameters relate the orientation and the position of the cameras w.r.t. each other. While intrinsic parameters usually need to be estimated once for a given camera (unless the camera has a variable focal length), the extrinsic parameters must be recomputed whenever cameras are moved or reoriented (on purpose or accidentally). Also, intrinsic calibration can be performed in the lab, before deploying the cameras, whereas extrinsic calibration is only possible after deployment. Especially in the case of temporary installations, e.g., to capture public events, the available time for calibration at deployment time is often limited and an efficient and reliable procedure is needed.

In this paper, we assume that the intrinsic parameters are known and we focus on extrinsic calibration.

Most existing extrinsic calibration methods first find distances to calibration objects by locating image features from different cameras. These features correspond to the same physical location on an object. Then the distances to the camera of these object points are computed using epipolar geometry from the correspondences. Finally an optimization routine simultaneously refines these estimates and computes the best matching calibration parameters. The most popular methods for the optimization step are generic non-linear optimisation techniques, or algebraic techniques based on the rank-4 factorization of an “observation matrices” [1]. All of these methods tend to get stuck in local optima unless they are initialised with good estimates for the distances to the cameras.

A general problem is that robustly finding correspondences is not trivial because features may be confused with other features, resulting in outliers; some features may not have correspondences due to occlusions. In practice, occlusions of *points on objects* are difficult to avoid, e.g., a checker board pattern cannot be seen well from directions parallel to the checker board (or from behind the checker board).

In this paper, we propose a novel extrinsic calibration method using a luminescent sphere as the calibration object. Spheres have several advantages over the typical point-like calibration objects:

- Spheres have consistent appearance when observed from an arbitrary direction. Therefore the center of the sphere can be used as a *feature point*, rather than specific points on the calibration object. This means use of spheres can improve locating image features which will benefit classical point-like features-based method. While the sphere center is not visible directly, it can be estimated from an image, and correspondences between camera views can be found even if the cameras observe different sides of the sphere. In principle this is even possible for partially occluded spheres (but we will not treat that case in this paper).
- As the sphere is a large and easy recognizable object, finding corresponding spheres in various views is quite easy, even in images of low resolution. As the sphere is luminescent, simple intensity thresholding suffices to locate the spheres in the images.
- Assuming the sphere size is known, it is possible to estimate the distance to a specific camera, solely based on the observed image, *i.e.*, without using information from other cameras. This means we can avoid using epipolar geometry to determine these distances, which are needed in the optimization method.

As we can estimate the 3D position of the sphere center w.r.t. the local camera coordinate system, we propose to use a less known method [2] which is based on orthogonal procrustes analysis to get the initial estimate of pairwise extrinsic parameters. Then, we propose to apply an optimization method which was developed by Bouguet [3], to jointly refine the extrinsic parameters for all cameras. The optimization step minimizes the total reprojection error (see Section 6) of calibration samples over all the extrinsic parameters.

The first contribution of the paper is that we propose a simple and accurate method to estimate the 3D position of the sphere center w.r.t. the local camera coordinate system. Our method requires only the estimate of the center of the projected sphere and its area. It is simpler and more robust against image segmentation error than other sphere-based calibration methods which process the sphere images by tracing the contour of the elliptical projection of the sphere.

In Section 7.2, we show that our method estimates the 3D positions of sphere centers w.r.t. the camera coordinate system more accurately than the sphere based method [4], which is one of the few methods designed for a similar purpose. Specifically the distance error of our method is smaller than the error of the sphere based method [4].

While the sphere locations are only used as intermediate results, a higher accuracy in these sphere locations still leads to a more accurate and stable estimation of extrinsic parameters. Specifically in Section 7.2 we will show that our method provides more accurate scale information. What is more, the projection, the triangulation and the reprojection error of our method are smaller than the error of the sphere-based method [4].

A second contribution is that we experimentally show that the orthogonal procrustes analysis achieves more accurate and stable calibration compared to the rank-4 factorization method. Specifically, in Section 7.3 we will show that the projection, the triangulation and the reprojection error of orthogonal procrustes are always smaller than the rank-4 factorization method. Moreover, the rank-4 factorization method requires at least 4 non-coplanar training samples, while orthogonal procrustes approach just needs 3 non-collinear training samples.

The final contribution is that we compare our algorithm with a popular reference calibration method of Svoboda *et al.* [5] and show that our method outperforms. Their method firstly estimates the projective depth using the method of Sturm *et al.* [1], which exploits epipolar geometry. Then, it computes projective structures via rank-4 factorization and does Euclidean stratification based on the concept of the absolute conic [6,7]. Finally, it applies the bundle adjustment [8] to refine the calibration. Their method requires at least 8 training samples (non-coplanar) and 3 cameras, which makes it not applicable to calibration of a camera pair. Moreover, they cannot provide scale information without metric measurements.

In Section 7.3, we will show that the projection and the triangulation error of our method are always smaller than the errors in the method of Svoboda. When considering reprojection error, the error of our method is slightly larger (3%) than the error of Svoboda’s method. Moreover, we will also show that the image of three sphere locations suffices for accurate calibration. While the method of Svoboda *et al.* requires at least eight distinct non-coplannar positions. An additional benefit of our approach is that it can extrinsically calibrate two cameras at a time, whereas the method of Svoboda requires at least three cameras.

The remainder of this paper is organized as follows. Section 2 gives a survey of works in the literature for extrinsic calibration. Section 3 provides details about intrinsic and extrinsic parameters. Section 4 explains how to estimate 3D positions of sphere centers w.r.t. the camera coordinate system. Section 5 discusses the actual extrinsic calibration procedure based on the aforementioned 3D positions’ estimation. In Section 6, we explain three criteria for accuracy evaluation of camera calibration techniques. Section 7 shows the experimental results. Finally, Section 8 concludes the paper.

## 2. Related Work

In recent years, extrinsic calibration of camera networks was well studied both in the photogrammetric and the computer vision community.

Many calibration methods have been proposed to do both intrinsic and extrinsic calibration using a calibration object with known world coordinates [9,10,11,12]. Such a calibration object consists of several easily detectable non-coplanar feature points with known relative 3D positions. Point correspondences between 3D world points and image points would be established by fixing the world coordinate system in the calibration object. Then, these algorithm estimate both intrinsic and extrinsic parameters (w.r.t. the fixed world coordinate system) which best map picture coordinates to corresponding 3D world coordinates. Calibration of a single camera can be done very efficiently for these methods. But for calibration of a camera network, it is tedious and cumbersome to calibrate all the cameras simultaneously using these reference objects, as it is often extremely difficult to make all the points on the calibration object simultaneously visible in all views. Moreover, it involves the design and use of some highly accurate tailor-made calibration patterns, which are often difficult and expensive to manufacture.

Zhang [13] proposed a calibration algorithm which uses a planar grid pattern as the calibration object. This method is mainly for intrinsic calibration. It can also do the extrinsic calibration for a stereo case with short baseline as soon as the planar pattern can be simultaneously visible in both cameras. Ueshiba *et al*. [14] proposed a similar method which also relies on the planar pattern. Patterns required for these methods [13,14] are easy and cheap to manufacture, which makes them flexible. However, for extrinsic calibration of a camera network or stereo case with wide baseline, these algorithms also encounter the problem of simultaneous visibility.

One way to avoid the problem of occluded features of a calibration object is to create a virtual calibration object by simply moving a detectable point through the working volume. Svoboda *et al.* proposed an approach with less user interaction for intrinsic and extrinsic calibration of camera networks [5]. They use a moving laser pointer emitting a bright spot. By waving the bright spot in front of the cameras a large number of correspondences of the virtual calibration object can be detected. As the laser pointer needs to be moved many times, it is not easy to avoid occlusions in all camera views simultaneously. In contrast, sphere-based methods can use a much smaller number of measurements, which makes it practical to avoid occlusions by moving away from the sphere. As the laser pointer is moved, the method of Svoboda *et al.* also requires accurate time synchronization between cameras to avoid erroneously associating feature points corresponding to changes of laser pointer positions.

Aslan *et al.* developed a similar approach to automatically calibrate the extrinsic parameters of multiple cameras [15]. Instead of a bright spot, they detected people walking through the room and used a reference point on top of the person’s head as the calibration feature. The relative pose of every camera pair is estimated using the corresponding feature points, and with this, the complete camera network is built up using a global error minimization technique. As there is no unique detectable point on top of a person’s head, different cameras will always detect different feature points, which causes problem for points correspondences. In contrast, we propose to use a sphere which has consistent appearance when observed from an arbitrary direction.

Sinha *et al*. used dynamic silhouettes of a moving person to fully calibrate a synchronized multi-camera system [16]. Their algorithm first uses silhouettes to estimate a set of fundamental matrices, which are then applied to compute a projective calibration. The projective calibration is upgraded to a Euclidean one using the self-calibration technique. Finally they apply the bundle adjustment to improve the solution. As their calibration approach relies on silhouettes, it requires a good silhouette extraction method for calibration data capturing. Moreover, the silhouette of a person changes when the person moves, which will cause correspondence problem.

Spheres have been considered as calibration objects since they have consistent appearance when observed from an arbitrary direction. Many sphere-based camera calibration approaches have been proposed. Some of them are only for intrinsic calibration [17,18,19,20,21].

Agrawal *et al.* [4] and Zhang *et al.* [22] proposed to do both intrinsic and extrinsic calibration by putting a sphere at three or more different places. For extrinsic calibration, both methods first estimate the 3D positions of the sphere centers in each of the camera coordinate systems based on known intrinsic parameters and conics of the ellipse (projection of a sphere in the image). Then, they obtained the relative rotation and translation between two cameras by 3D point registration of the estimated sphere centers. The accuracy of both camera calibration methods depends highly on the accuracy of the conic matrices which can be obtained by ellipse fitting of the projection of the sphere in the image. Thus, the quality of ellipse detection strongly affects the accuracy of calibration results. In contrast, our method requires only the estimate of the center of the projected sphere and its area. These are simpler to compute and more robust against errors in the projected sphere’s estimated contours.

## 3. Preliminaries

Extrinsic parameters are expressed with respect to a reference coordinate system, which is also called the world coordinates system. Since we have multiple cameras, we will associate a distinct world coordinate system in which a point is denoted as $r^{w}={\left(X_{W},Y_{W},Z_{W}\right)}^{T}$ , where the superscript *T* denotes matrix transposition. In the camera coordinate system, a point is denoted as $r^{\left(k\right)}={\left(X^{\left(k\right)},Y^{\left(k\right)},Z^{\left(k\right)}\right)}^{T}$ with each camera *k*. The world coordinates **r**^*w*^ are w.r.t. a single reference coordinate system. Without loss of generality, we choose the coordinate system of the first camera as the world coordinate system: **r***^w^* = **r**^(1)^. The camera coordinates **r**^(*k*)^ are related to the world coordinates **r**^*w*^ by $r^{\left(k\right)}=R^{\left(k\right)}r+c^{\left(k\right)}$, where **c**^(*k*)^ are the coordinates of the origin of the global world coordinate system within the local coordinate system of camera *k* and *R*^(*k*)^ is a 3×3 rotation matrix. The purpose of this paper is to find **c**^(*k*)^ and *R*^(*k*)^ for each camera.

Intrinsic parameters define the imaging geometry of the camera. Since we deal with only one camera for intrinsic parameters, we will drop the superscripts *k*. In the camera coordinate system, a point is denoted as $r={\left(X,Y,Z\right)}^{T}$. We assume that the camera is modelled by the usual pinhole, its coordinate system has its origin in the optical center of the camera, its *Z* axis (optical axis) perpendicular to the image plane. Therefore, the image plane is given by Z=f, where *f* is the focal length of the camera in physical units (e.g., centimeter). Moreover in each camera image the projection of a 3D point can be characterized by its normalized image coordinates x=(x,y) which have the same physical units as the camera coordinates r. Alternatively, a projected point can also be identified by its integer pixel coordinates $u={\left(u,v\right)}^{T},$ *i.e.*, a column and row number in the image.

Assuming a zero-skew camera, the pixel and normalised image coordinates are related by

(1) $$\begin{array}{c} \left[\begin{array}{c} u\\ v\\ 1 \end{array}\right]=\left[\begin{array}{ccc} \frac{1}{s_{x}} & 0 & u_{0}\\ 0 & \frac{1}{s_{y}} & v_{0}\\ 0 & 0 & 1 \end{array}\right]\left[\begin{array}{c} x\\ y\\ 1 \end{array}\right] \end{array}$$

in which $s_{x},s_{y}$ is the pixel dimensions in physical units. We assume that intrinsic calibration has already been performed. This means we know $f_{x},f_{y}$ , and the optical center position $\left(u_{0},v_{0}\right)$. Moreover, $f_{x}\;=\;f/s_{x},f_{y}=f/s_{y}$ are then related to the focal distance *f*.

As such three specific coordinate systems are associated with each camera observation of a point. The first set of coordinates $r={\left(X,Y,Z\right)}^{T}$ is expressed in physical units and indicate the 3D position of a point relative to the camera. The second set and third sets of coordinates (x,y) are the normalized image coordinates expressed in physical units and the coordinates (u,v), expressed in pixels, respectively. We also have the relationship:

(2) $$\begin{array}{c} Z\left[\begin{array}{c} x\\ y\\ 1 \end{array}\right]=\left[\begin{array}{ccc} f & 0 & 0\\ 0 & f & 0\\ 0 & 0 & 1 \end{array}\right]\left[\begin{array}{c} X\\ Y\\ Z \end{array}\right] \end{array}$$

Combining Equations (1) and (2) we get

(3) $$\begin{array}{c} Z\left[\begin{array}{c} u\\ v\\ 1 \end{array}\right]=\left[\begin{array}{ccc} f_{x} & 0 & u_{0}\\ 0 & f_{y} & v_{0}\\ 0 & 0 & 1 \end{array}\right]\left[\begin{array}{c} X\\ Y\\ Z \end{array}\right] \end{array}$$

So we can estimate the 3D position of a point w.r.t. the camera coordinate system if we know *Z*, (u,v) and all 4 intrinsic parameters. In Section 4, we will show how to estimate *Z* based on the projection of a sphere in the image.

## 4. Estimating the Sphere Centers in a Local Camera Coordinate System

### 4.1. Projection of a Sphere on the Camera Plane

In this section, we assume that f=1 for simplifying the derivation. Consider a sphere with radius $R_{s}$ with its center at position $r_{s}$, where $r_{s}={\left(X_{s},Y_{s},Z_{s}\right)}^{T}$ is w.r.t. the camera coordinate system. The center is located at a distance $d_{s}$ from the origin (optical center of the camera). We also assume that the size of the sphere is small compared to the distance to the camera plane. Another assumption is that the center of the sphere projects to the center of the ellipse.

**Figure 1:**
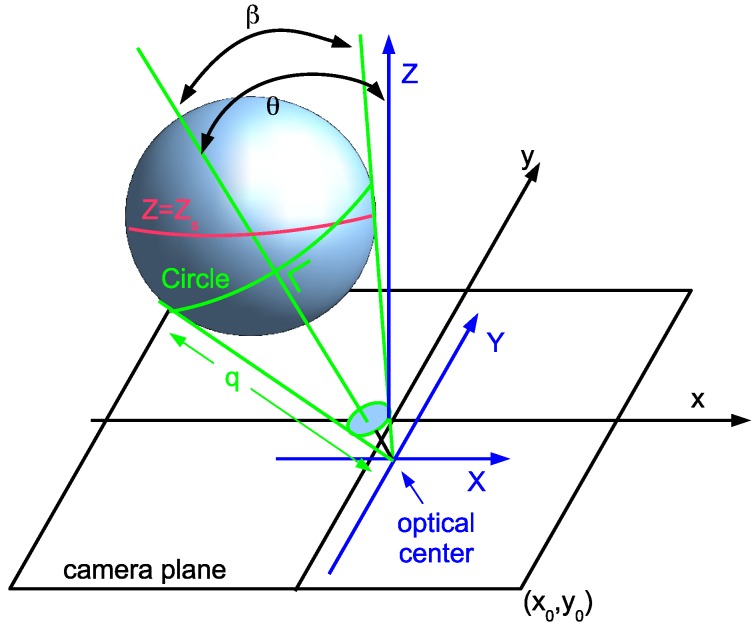
Projection of a sphere.

Figure 1 shows that the sphere is projected as an ellipse on the camera plane. In fact the ellipse outline is the projection of a very specific circle (indicated in green) on the sphere. The circle is also the intersection of a cone (also shown in green) tangent to the sphere and the sphere itself. The cone has an opening angle *β*. The center of the circle is $r_{c}={\left(X_{c},Y_{c},Z_{c}\right)}^{T}$ equals $d_{c}e_{n}$ and is at a distance $d_{c}$ from the origin. In general, the radius $R_{c}$ of the circle does not equal the radius $R_{s}$ of the sphere and $d_{c}\neq d_{s}$.

We rotate the camera coordinate system along the *Y* axis by an angle of -θ, then along the *Z* axis by an angle of 1.5π+ϕ. We will get three orthogonal unit vectors $e_{1}$, $e_{2}$ and $e_{n}$ which define a right-handed coordinate system. $e_{n}$ is the unit vector pointing in the direction of the sphere:

$$e_{n}={\left[\begin{array}{ccc} \sin\phi \sin\theta , & \cos\phi \sin\theta , & \cos\theta \end{array}\right]}^{T}$$

The other two unit vectors are

$$e_{1}={\left[\begin{array}{ccc} \sin\phi \cos\theta , & \cos\phi \cos\theta , & -\sin\theta \end{array}\right]}^{T}$$

and

$$e_{2}={\left[\begin{array}{ccc} -\cos\phi , & \sin\phi , & 0 \end{array}\right]}^{T}$$

We define *q* as the slant height of the cone. Taking into account that the cone is tangent to the sphere, we have $\sin\beta =R_{s}/d_{s}$ and $q^{2}=d_{s}^{2}-R_{s}^{2}$. Taking into account that the circle is perpendicular to the axis of the cone we have $\tan\beta =R_{c}/d_{c}$, $d_{c}=q\cos\beta$ and $R_{c}=q\sin\beta .$

Therefore we can get $R_{c}$ and $d_{c}$ in terms of $R_{s}$ and *β*:

(4) $$R_{c}=R_{s}\cos\beta$$

(5) $$d_{c}=R_{s}/\sin\beta -R_{s}\sin\beta$$

If we introduce a parameter *α*, then the 3D position of points on that specific circle is given by

(6) $$r=d_{c}e_{n}+R_{c}\cos\alpha e_{1}+R_{c}\sin\alpha e_{2}\;\text{with}\;\alpha \in \left[0,2\pi \right]$$

In the camera plane, we have normalized image coordinates (x,y). In these coordinates, by using Equation (2) with f=1, the projected ellipse of that circle is given by

(7) $$\begin{array}{c} x=\frac{-R_{c}\sin\alpha \cos\phi +R_{c}\cos\alpha \sin\phi \cos\theta +d_{c}\sin\theta \sin\phi }{-R_{c}\sin\theta \cos\alpha +d_{c}\cos\theta } \end{array}$$

(8) $$\begin{array}{c} y=\frac{R_{c}\sin\alpha \sin\phi +R_{c}\cos\phi \cos\alpha \cos\theta +d_{c}\cos\phi \sin\theta }{-R_{c}\sin\theta \cos\alpha +d_{c}\cos\theta } \end{array}$$

In this equation, *α* varies from 0 to 2π, but all other variables are fixed.

We now rotate the image coordinate system to get new coordinates

(9) $$\begin{array}{c} x_{1}=x\cos\phi -y\sin\phi \end{array}$$

(10) $$\begin{array}{c} y_{1}=x\sin\phi +y\cos\phi \end{array}$$

We change once again to a new coordinate system, but this time using a translation instead of a rotation. The new coordinates are $\left(x_{1},y_{2}\right)$ with $y_{2}=y_{1}+\Delta$, in which

(11) $$\Delta =\frac{R_{c}^{2}\sin\left(\theta \right)\cos\left(\theta \right)+d_{c}^{2}\sin\left(\theta \right)\cos\left(\theta \right)}{R_{c}^{2}\sin^{2}\left(\theta \right)-d_{c}^{2}\cos^{2}\left(\theta \right)}$$

Then we obtain the ellipse equation which only has terms in $x_{1}^{2}$, $y_{2}^{2}$ and of course the constant term 1, *i.e.*, it is of the form

(12) $${\left(\frac{x_{1}}{\lambda_{x}}\right)}^{2}+{\left(\frac{y_{2}}{\lambda_{y}}\right)}^{2}=1$$

From this we can deduce that the ellipse is centered at $\left(x_{1},y_{2}\right)=\left(0,0\right)$, which corresponds to $x_{1}=0$ and

(13) $$y_{1}=-\Delta =-\frac{R_{c}^{2}\sin\left(\theta \right)\cos\left(\theta \right)+d_{c}^{2}\sin\left(\theta \right)\cos\left(\theta \right)}{R_{c}^{2}\sin^{2}\left(\theta \right)-d_{c}^{2}\cos^{2}\left(\theta \right)}$$

In fact, $y_{1}$ is also the distance of the center of the ellipse to the center of the camera image plane, expressed in the original (non-translated, non-rotated) image coordinates (x,y). By replacing $R_{c}$ and $d_{c}$ using Equations (4) and (5), we can get $\Delta ,\lambda_{x}^{-2},\lambda_{y}^{-2}$.

Then we introduce the variable $\epsilon =R_{s}/Z_{s}$. This variable is typically small because the size of the sphere is assumed small compared to the distance to the camera plane. Note that also ϵ=sinβ/cosθ. Using the fact that *ϵ* is small, we can employ Taylor series approximation underneath the square roots and find that

(14) $$-\Delta =\epsilon^{2}\tan\theta +\tan\theta \approx \tan\theta$$

(15) $$1/\lambda_{x}=\frac{\sqrt{-\epsilon^{2}+1}}{\epsilon }\approx \frac{1}{\epsilon }$$

and

$$1/\lambda_{y}=\frac{\sqrt{\epsilon^{2}\cos^{4}\theta -2\epsilon^{2}\cos^{2}\theta +\cos^{2}\theta }}{\epsilon }\approx \frac{\cos\theta }{\epsilon }$$

We know that the area of the ellipse is $A=\pi \lambda_{x}\lambda_{y}$, we also know that $\epsilon =R_{s}/Z_{s}$ and ϵ=sinβ/cosθ. Combining these equations with Equations (14), (15) and (16), we can deduce that $Z_{s}\;=\;R_{s}/\epsilon =\;R_{s}\sqrt{\pi /(A\cos\theta ))}$. This means if we know area *A* and *θ*, we can get the estimated $Z_{s}$ with known $R_{s}$. In Section 4.2, we will give the procedure about how to estimate *A* and *θ* from an image of a sphere and known intrinsic parameters.

### 4.2. Estimation of the Sphere Position from an Image

Based on the aforementioned derivation, the practical procedure to estimate the center $r_{s}$ of the sphere from the camera picture works as follows:

1. Segment the ellipse (projection of the sphere) within the image. This yields a binary image which resembles only an ellipse. In this process, we do not need to estimate the exact parameter of the ellipse, as we only need to estimate the the center and area of the projected sphere. In our experiments, we propose to use a lighted sphere, and slightly darkened the room, simple thresholding suffices to segment the ellipse blobs from the background.
2. Since we assume that the center of the sphere projects to the center of the ellipse, from the binary image we estimate the pixel coordinates $u_{s}$ and $v_{s}$ of the center of gravity of the binary blob which represents the ellipse in the segmented image. After that we can get $x_{s}=\left(u_{s}-u_{0}\right)/f_{x}$, and $y_{s}=\left(v_{s}-v_{0}\right)/f_{y}$. We have $-\Delta =\sqrt{x_{s}^{2}+y_{s}^{2}}$. From *Δ* we can compute θ=-arctanΔ.
3. Compute the area *A* of the (filled) projected ellipse: $A=N\left(s_{x}s_{y}\right)=N/\left(f_{x}f_{y}\right)$, where *N* is the number of pixels in the binary blob representing the segmented ellipse. This area also equals $A=\pi \lambda_{x}\lambda_{y}\approx \pi \epsilon^{2}/\cos\theta$. So $Z_{s}=R_{s}/\epsilon =R_{s}\sqrt{\pi /(A\cos\theta ))}$.
4. From $x_{s}$, $y_{s}$ and $Z_{s}$ compute $X_{s}=x_{s}Z_{s}$ and $Y_{s}=y_{s}Z_{s}$.

## 5. Multi-Camera Calibration

As stated in Section 3, a given point can be expressed in world coordinates $r^{w}=\;(X_{W},Y_{W},Z_{W})$ or in camera-*k* specific camera coordinates **r**^(*k*)^. The coordinate systems are related as $r^{\left(k\right)}=(X^{\left(k\right)},Y^{\left(k\right)},Z^{\left(k\right)})=R^{\left(k\right)}r^{w}+c^{\left(k\right)}$, where **c**^(*k*)^ are the coordinates of the origin of the global world coordinate system within the local coordinate system of camera *k* and *R*^(*k*)^ is a 3×3 rotation matrix. Note that *R*^(*k*)^ is an orthogonal matrix, so ${\left(R^{\left(k\right)}\right)}^{T}R^{\left(k\right)}=I$, with *I* the identity matrix. The goal is to find **c**^(*k*)^ and *R*^(*k*)^ for each camera *k*. Using the equations in Section 4, we can estimate the camera coordinates of the sphere center from the image of camera *k*. In our implementation, we select the coordinate frame of camera 1 as the world coordinate frame, which means **r**^*w*^ = **r**^(1)^, *R*^(1)^=I and **c**^(1)^ = **0**. The remaining problem is then to estimate *R*^(*k*)^ and **c**^(*k*)^ for all other cameras. This problem is equivalent to computing the 3D rigid body transformation which optimally aligns two sets of points for which the correspondence is known. Eggert *et al.* [23] presented a comparative analysis of four popular and efficient algorithms, each of which computes the translational and rotational components of the transform in closed form, as the solution to a least squares formulation of the problem. Eggert *et al.* [23] proved that the method [2] provides the best overall accuracy and stability. The first solution [2] was developed by Arun *et al.* and is based on computing the singular value decomposition of a matrix derived from the standard representation. So we propose to use the method of Arun *et al.* [2], which investigates the solution of orthogonal procrustes analysis [24]. For comparison with the commonly used rank-4 method, we also implement the rank-4 factorization method of Sturm *et al.* [1].

### 5.1. Extrinsic Calibration based on Rank-4 Factorization

In the following we switch to homogeneous coordinates to perform the multi-camera calibration. We use $\tilde{x}$ to denote the augmented vector by adding 1 as the last element: $r^{\left(1\right)}={\left(X^{\left(1\right)},Y^{\left(1\right)},Z^{\left(1\right)},1\right)}^{T}$. In this case, the relationship between the two camera coordinates can be written compactly as

(16) $$r^{\left(k\right)}=P_{k}r^{\tilde{(}1)}$$

where

(17) $$P_{k}=\left[R^{\left(k\right)},|,c^{\left(k\right)}\right]$$

Suppose the calibration sphere is moved to *N* different positions in the room. At each position, all cameras capture images of the sphere and we get the estimated camera coordinates $r_{i}^{\left(1\right)}$ and $r_{i}^{\left(k\right)}$, with i=1,2...N. Let $F^{\left(1\right)}$ be the matrix with columns $r_{i}^{\tilde{(}1)}$ and $F^{\left(k\right)}$ be the matrix with columns $r_{i}^{\left(k\right)}$, then we have

(18) $$F^{\left(k\right)}=P_{k}F^{\left(1\right)}$$

As the rank of $F^{\left(k\right)}$ is at most 4 and this provides a means to compute the matrices *P_k_* using the rank-4 factorization method [1]. The calibration proceeds as follows:

1. Let $F^{\left(k\right)}=USV^{T}$ be the SVD decomposition of $F^{\left(k\right)}$; extract $U_{1}$, the first 4 columns of the matrix *U*.
2. At this point, we define $A\overset{\Delta }{=}{\left(U_{1}^{T}P_{k}\right)}^{-1}$, and $D=U_{1}^{T}F^{\left(k\right)}$. Combining *A*, *D* and Equation (18), we know that AD must equal $F^{\left(1\right)}$, from which we can compute *A*.
3. Finally we compute $P_{k}=U_{1}A^{-1}$.

As rank-4 factorization is applied here, it requires N>3 and the sphere positions to be non-coplanar. Due to noise in the data, the estimated matrix *R*^(*k*)^ in *P_k_* does not generally satisfy the properties of a rotation matrix. Therefore, we use the orthogonal procrustes solution presented in Section 5.2 to make the estimated rotation matrix orthogonal, then we recalculate the translation parameters.

### 5.2. Extrinsic Calibration based on Orthogonal Procrustes

Arun *et al.* [2] proposed to estimate *R*^(*k*)^ based on orthogonal procrustes analysis. The orthogonal procrustes problem is the following problem in matrix algebra: given matrices *A* and *B*, find a square orthogonal matrix *Q* such that QA is as close as possible to *B*: specifically *Q* is defined as ${\text{arg min}}_{Q^{\prime }}∥Q^{\prime }A-B∥$, where ∥.∥ is the Frobenius matrix norm. In other words, the minimization is performed in the sense of the total least squares. This problem was originally solved by Peter Schonemann in 1964 and the solution was later published [24]. This problem is equivalent to finding the nearest orthogonal matrix *Q* to a given matrix $M=A^{T}B$. In terms of the singular value $M=USV^{T}$ of *M*, this is given by $Q=UV^{T}.$

As we know that $r_{i}^{\left(k\right)}=R^{\left(k\right)}r_{i}^{\left(1\right)}+c_{i}^{\left(k\right)}$, with i=1,2...N. In order to solve this problem using orthogonal procrustes, we need to decouple the translation and rotation. We calculate the centroid of $r_{i}^{\left(1\right)}$ and $r_{i}^{\left(k\right)}$ using

(19) $$\begin{array}{ccc} \bar{r^{\left(1\right)}}=\frac{1}{N}\sum_{i=1}^{N}r_{i}^{\left(1\right)}, & & \bar{r^{\left(k\right)}}=\frac{1}{N}\sum_{i=1}^{N}r_{i}^{\left(k\right)} \end{array}$$

Let $H^{\left(1\right)}$ be the matrix with columns $r_{i}^{\left(1\right)}-\bar{r^{\left(1\right)}}$, i=1,2...N. and $H^{\left(k\right)}$ be the matrix with columns $r_{i}^{\left(k\right)}-\bar{r^{\left(k\right)}}$, then we have $H^{\left(k\right)}=R^{\left(k\right)}H^{\left(1\right)}$, or equivalently ${H^{\left(k\right)}}^{T}={H^{\left(1\right)}}^{T}{R^{\left(k\right)}}^{T}.$ Using orthogonal procrustes we decompose $H^{\left(1\right)}{H^{\left(k\right)}}^{T}$ as $H^{\left(1\right)}{H^{\left(k\right)}}^{T}=U_{2}S_{2}V_{2}^{T}.$ As pointed out by Arun *et al.*, there will be three possibilities for the solution of *R*^(*k*)^ from geometrical considerations.

1. $\left\{r_{i}^{\left(k\right)}\right\}$ are not coplanar. In this case, the rotation solution is unique, $R^{\left(k\right)}=V_{2}U_{2}^{T}$ is the desired solution.
2. $\left\{r_{i}^{\left(k\right)}\right\}$ are coplanar but not collinear. There is a unique rotation as well as a unique reflection. In this case, we just need to check the value of $det(V_{2}U_{2}^{T})$: $R^{\left(k\right)}=V_{2}U_{2}^{T}$ if $det(V_{2}U_{2}^{T})=1$, $R^{\left(k\right)}=V_{2}^{{}^{\prime }}U_{2}^{T}$ if $det(V_{2}U_{2}^{T})=-1$, where $V_{2}^{{}^{\prime }}$ is obtained by changing the sign of the last column of matrix $V_{2}$.
3. $\left\{r_{i}^{\left(k\right)}\right\}$ are collinear. There will be no unique solution for *R*^(*k*)^.

Once we obtain the rotation matrix *R*^(*k*)^, the translation vector **c**^(*k*)^ is then given by

(20) $$\begin{array}{c} c^{\left(k\right)}=\bar{r^{\left(k\right)}}-R^{\left(k\right)}\bar{r^{\left(1\right)}} \end{array}$$

### 5.3. Refinement through Gradient Descent

The equations in Section 5.1 and Section 5.2 provide a quite good estimate of the extrinsic calibration parameters, but their accuracy is limited by the approximation we made during estimating the sphere position w.r.t. the local camera coordinate system in Section 4. Moreover, they are not jointly optimised since we do pairwise calibration to relate each camera to the first camera. To jointly refine the extrinsic parameters for all cameras, we apply an optimization method which was developed by Bouguet [3] to optimize the results. The optimization step minimizes the total reprojection error of training samples (see Section 6) over all the extrinsic parameters. The objective function to be minimized is the mean-square discrepancy between the observed ellipse center in the image and their image reprojections computed using the estimated extrinsic calibration matrices. The optimization is implemented using an iterative gradient descent procedure.

### 5.4. Alignment with a World Coordinate System

The aforementioned calibration yields the external camera parameters in the coordinate frame of the first camera. In practical applications, it is often desirable to have all parameters in some user specified world coordinate system. For example, for camera networks which are intended for indoor people tracking, we would like to have the Z=0 plane to coincide with the ground floor. For that, and only for that, we need ground truth measurements of at least three sphere centers w.r.t. the user-specified world coordinate system. Then, we use the procrustes approach of Section 5.2 to compute the transform between the user-specified coordinate system and camera-1’s coordinate system.

## 6. Performance Measures

There exist several evaluation methods and accuracy measures to compare calibration methods. In the following, we assume that we have acquired *m* images of *n* additional spheres which are used only as test samples, but not to estimate *R*^(*k*)^ and **c**^(*k*)^. We also assume that the ground truth coordinates of these test samples are known. In practice, we measure them using a tape measure according to the user specified world coordinate system. We denote by d(a,b) the Euclidean distance between two points a and b in 3D space.

1. *δ***r**^*w*^. The *triangulation error* is a measurement of how the calibration matrices influence the accuracy of multi-camera triangulation. Let $r_{i}^{w}$ be the ground truth position of the *i*th test sample, and ${\hat{r}}_{i}^{w}$ its position estimated using triangulation [25] based on estimated extrinsic parameters and image positions of the sample. This is also the classical method for 3D reconstruction, it represents how well we can measure the 3D world with estimated extrinsic parameters. The estimated 3D positions ${\hat{r}}_{i}^{w}$ will differ from the true positions $r_{i}^{w}$ not only because of measurement errors but also due to any inaccuracies in the calibration matrices and the process of triangulation. The triangulation error *δ***r**^*w*^ pools all of these errors. As such it is not an absolute measure of calibration accuracy. However, as we use the same image feature points and the same triangulation method to compare various calibration methods, *δ***r**^*w*^ is a good quality measure for the extrinsic calibration. The error is expressed in in physical units (e.g., centimeter) and is defined as
  (21) $$\delta r^{w}=\frac{1}{n}\sum_{i=1}^{n}d\left(r_{i}^{w},{\hat{r}}_{i}^{w}\right)$$
2. $\delta u^{pr}$. The *projection error* is a measurement of how the calibration matrices influence the accuracy of projections of 3D points on image planes. Let $u_{ij}$ be the observed pixel coordinates of the *i*th sample in the *j*th camera’s image, while ${\hat{u}}_{ij}^{pr}$ is the estimated position through projection. When image feature points are projected from 3D points (with known position), the estimated 2D image positions ${\hat{u}}_{ij}^{pr}$ will differ from the true positions $u_{ij}$ not only because of measurement errors, but also due to any inaccuracy in the calibration matrices. The projection error $\delta u^{pr}$ takes all these errors into account. So it is not an absolute measurement of the calibration accuracy. However, since the same image feature points are used to compare different calibration methods, $\delta u^{pr}$ is another good quality measurement for the extrinsic calibration. The error is expressed in pixels and is defined as
  (22) $$\delta u^{pr}=\frac{1}{nm}\sum_{i=1}^{n}\sum_{j=1}^{m}d\left({\hat{u}}_{ij}^{pr},u_{ij}\right)$$
3. $\delta u^{repr}$. The *reprojection error* is a measurement of self-consistency of the calibration matrices. Different from the projection error, the 3D points are firstly obtained from triangulation based on estimated extrinsic parameters and image points. Then, image feature points are projected from these 3D points. The estimated 2D image positions ${\hat{u}}_{ij}^{repr}$ will differ from the true positions $u_{ij}$ not only because of measurement errors, but also due to any inaccuracy in the calibration matrices and the process of triangulation. The reprojection error $\delta u^{repr}$ takes all these errors into account. So it is not an absolute measurement of the calibration accuracy. However, since the same image feature points are used to compare different calibration methods, $\delta u^{repr}$ is another good quality measure for the extrinsic calibration. The error is expressed in pixels and is defined as
  (23) $$\delta u^{repr}=\frac{1}{nm}\sum_{i=1}^{n}\sum_{j=1}^{m}d\left({\hat{u}}_{ij}^{repr},u_{ij}\right)$$

## 7. Experiments and Results

### 7.1. Dataset for Evaluation

For evaluation, we calibrated a multi-camera tracking system composed of four side view cameras using a 25 cm diameter “globe lamp” sold as a garden light. The cameras were mounted at a height of about 3 m in each corner of a room (8.6 m by 4.8 m) with a resolution of 780 by 580 pixel. These cameras were firstly intrinsically calibrated using the method of Zhang [13].

**Figure 2:**
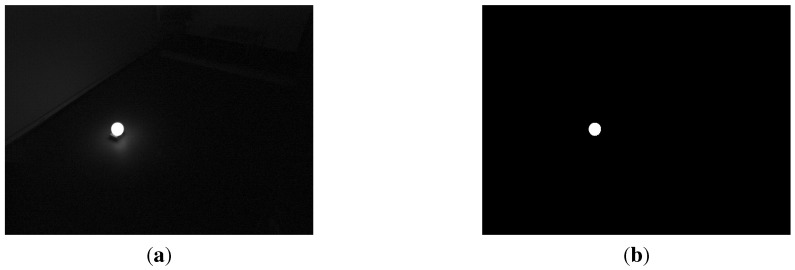
Images used for calibration. (**a**) Original captured image; (**b**) Binary image after simple thresholding

The practical calibration proceeds as follows:

1. Put the sphere in the common overlapping area at minimum 3 distinct non-collinear locations and let each camera capture an image at each location. The spheres are distributed evenly over working volume of the multi-camera system.
2. As we use a lighted sphere, and slightly darkened the room, simple thresholding suffices to segment the sphere blobs from the background. Figure 2a is an example of captured images of the sphere. Figure 2b is the image which only shows the ellipse after simple thresholding.
3. Estimate the center of mass and the area of the binary blobs. Then, compute the estimated sphere position w.r.t. the camera coordinate system based on the equations in Section 4.
4. Compute the extrinsic calibration matrices according to the equations in Section 5.

To assess the robustness against the number and distribution of training samples, we will show results for various numbers of calibration (training) samples. These are randomly selected from a set of 45 training samples (*i.e.,* four images of the sphere in 45 different positions). Some of the performance criteria in Section 6 require ground truth expressed in the user specified world coordinate system. For this purpose we measured the positions of 10 sphere centers using a tape measure. As it is not possible to know where exactly the center of the sphere is, we put the sphere on top of a cube box whose edge length is the same as the diameter of the sphere. So the position of the sphere center can be obtained by combining the position of the box corner with the diameter of the sphere. To test the accuracy of calibration, we also captured 40 more test samples. In this case, the sphere was placed in different positions than for the training samples. These test samples are never used to compute the calibration matrices, but only to estimate the accuracy of the matrices and the robustness of the proposed approach.

### 7.2. Comparison with Conics-based Sphere Calibration

In order to assess the accuracy of estimating sphere positions w.r.t. the camera coordinate system, we compared our method with the sphere-based method of Agrawal *et al.* [4]. This method performs both intrinsic and extrinsic calibration. For extrinsic calibration, they first estimate the positions of the sphere centers in each of the camera coordinate systems based on the intrinsic parameters and conics of the sphere projection. Then they obtain the relative rotation and translation between two cameras by registering the estimated two sets of sphere centers. For fair comparison, both methods use the same intrinsic parameters obtained using the method of Zhang [13].

**Figure 3:**
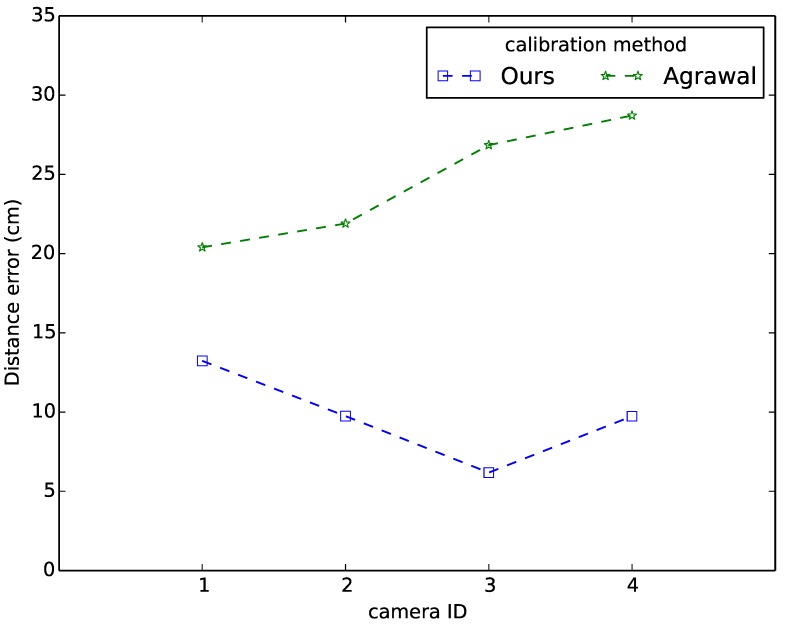
Mean distance error comparison.

As it is difficult to measure the ground truth position of a sphere w.r.t. the camera coordinate system, we propose to use the distance between the sphere center and the camera center as the evaluation criteria. For that we measure the position of each camera w.r.t. the predefined world coordinate system, we also have the positions of spheres w.r.t. the same world coordinate system, then we can calculate the euclidean distance. Meanwhile, we estimate the 3D positions of the sphere centers using our proposed approach, as well as Agrawal’s method. Then, we obtain an estimate of the distance between the sphere center and the camera center. We use all 40 test samples in this comparison. Figure 3 demonstrates that our approach provides more accurate and stable estimation of 3D positions of sphere centers compared to the conic-based method of Agrawal *et al.*, since the distance error of our method is 9.7 cm, while the average error of Agrawal’s method is 24.5 cm.

**Figure 4:**
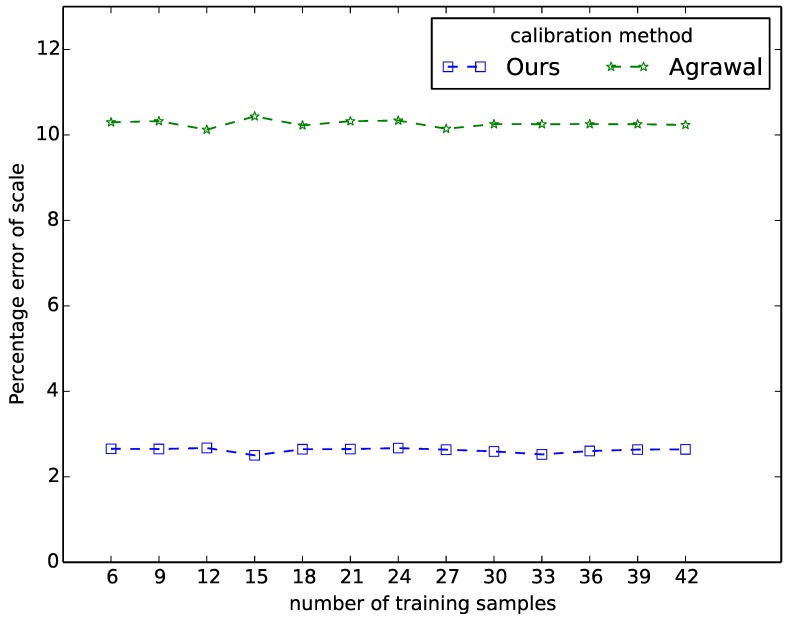
Scale error comparison with different number of training samples.

As both methods can estimate the distance between the spheres and the cameras, we can estimate the scale information (distance) between two 3D points which we define as *s*. Then we introduce another performance criterion: the *scale error* δs. For two test samples with positions $r_{1}^{w}$ and $r_{2}^{w}$ in the user-specified world coordinate system, we calculate the ground truth Euclidean distance between these two samples as $s_{w}=d\left(r_{1}^{w},r_{2}^{w}\right)$. After we obtain the extrinsic parameters of other cameras w.r.t. the coordinate system of the first camera, we can obtain the 3D positions (w.r.t. the coordinate system of the first camera) of the same two samples using triangulation based on the corresponding 2D image coordinates and estimated extrinsic parameters. Then we have $s_{c}=d\left(r_{1}^{\left(1\right)},r_{2}^{\left(1\right)}\right)$. This distance is computed in the camera coordinate system, but of course it should equal the ground truth distance in the absence of calibration and image processing errors. Then, the scale error is defined as $\delta s_{12}=\left|s_{c}-s_{w}\right|/s_{w}$. Since we have multiple test samples, we calculate the scale error for each combination of two samples, and calculate the average of all scale errors. Figure 4 shows that our approach allows more accurate and stable estimation of scale information compared to the conic based method of Agrawal *et al.* The scale error of our method is 2.4%, while the error of Agrawal’s method is 10.3%.

For those comparisons which require comparison to ground truth in the user-specified coordinate system, we use the same orthogonal procrustes analysis approach described in Section 5.2 to compute the transform to user-specified coordinates. To assess the robustness to the precise choice of training samples and the number *N* of training samples, we repeat the calibration procedure 100 times for each *N*, for various sets of training samples, randomly selected from the available ones.

We can conclude that our method outperforms the method of Agrawal *et al.* for estimating extrinsic parameters since the projection error, triangulation error and reprojection error of our method are nearly half of the errors of the method of Agrawal *et al.*, as illustrated in Table 1.

**Table 1:** Accuracy comparison between our method and the method of Agrawal *et al*. The first column represents the number of training samples. The numbers are listed as “a/s” where a is the average over 100 experiments and s is the standard deviation.

	$\delta u^{\mathrm{pr}}$(pixel)		*δ***r**^*w*^ (cm)		$\delta u^{\mathrm{repr}}$ (pixel)	
	Ours	Agrawal	Ours	Agrawal	Ours	Agrawal
06	6.2/3.6	12.3/3.0	5.1/2.3	8.5/2.2	4.2/3.4	9.8/2.5
09	5.3/1.1	11.6/2.0	4.6/0.6	8.3/1.6	3.4/1.0	9.2/1.5
12	5.2/0.8	10.9/1.5	4.6/0.4	8.1/1.3	3.2/0.6	8.6/1.0
15	5.0/0.5	11.0/1.3	4.5/0.3	8.3/1.2	3.0/0.4	8.5/0.9
18	5.0/0.6	10.7/1.0	4.5/0.3	8.1/0.9	3.0/0.4	8.4/0.6
21	4.9/0.4	10.7/1.0	4.5/0.2	8.1/0.9	2.9/0.3	8.3/0.7
24	4.9/0.3	10.6/0.8	4.5/0.2	8.1/0.8	2.8/0.2	8.2/0.5
27	4.8/0.3	10.3/0.6	4.4/0.2	7.9/0.6	2.7/0.2	8.1/0.4
30	4.8/0.2	10.4/0.5	4.5/0.1	8.0/0.5	2.7/0.1	8.1/0.3
33	4.8/0.2	10.4/0.5	4.4/0.1	8.0/0.5	2.7/0.1	8.1/0.3
36	4.8/0.1	10.4/0.4	4.4/0.1	8.0/0.4	2.7/0.1	8.1/0.3
39	4.8/0.1	10.3/0.3	4.4/0.1	8.0/0.3	2.6/0.1	8.0/0.2
42	4.7/0.1	10.3/0.2	4.4/0.1	8.0/0.2	2.6/0.1	8.0/0.1

### 7.3. Comparison with Epipolar Geometry Based Calibration

In order to show the feasibility and robustness of the proposed method, we also make a comparison to the calibration method of Svoboda *et al.* [5] using the same training and test samples.

Table 2, Table 3 and Table 4 show the comparison in terms of the projection error, the triangulation error and the reprojection error, respectively. It can be observed from these three tables that the accuracy of all methods improves by increasing the number of training samples. We can obtain that the orthogonal procrustes method is always more accurate and stable than the rank-4 method. We can also conclude that the refinement can improve the accuracy and stability of the orthogonal procrustes method. Compared to the method of Svoboda *et al.* in terms of the projection and the triangulation error, our refinement method is more accurate and stable, especially when the number of training samples is below 18. The reprojection error of our method is slightly larger (3%) than the error of Svoboda’s method. However, our refinement method can still produce reasonable result (the projection, the triangulation and the reprojection errors are 5.3/1.4,4.5/0.7,3.3/1.6, respectively), when we only have three training samples, wherever the method of Svoboda is not applicable.

In terms of practical aspect, it is required to crawl on the floor with a laser pointer for the method of Svoboda’s method in order to fill up the whole working volume. On the contrary, in our method we just need to put the sphere on the ground or on top of a box, which makes it more easy and pleasant to capture the calibration data.

**Table 2:** Projection error (pixel). The first column represent the number of training samples, and the first row gives the methods used for comparison. The refinement method uses the result of Orthogonal procrustes as initial guess, and then applies the proposed refinement method to improve the accuracy. The numbers are listed as “a/s” where a is the average over 100 experiments and s is the standard deviation

	Rank-4	Orthogonal Procrustes	Refinement	Svoboda
03	N/A	6.4/1.8	5.3/1.4	N/A
04	7.9/5.4	5.8/1.4	3.5/0.7	N/A
05	6.5/2.3	5.5/1.4	3.2/0.3	N/A
06	6.4/2.3	5.5/1.5	3.2/0.2	N/A
07	6.3/1.8	5.6/1.4	3.1/0.1	N/A
08	6.0/1.6	5.3/1.3	3.1/0.1	4.1/2.0
09	6.2/1.8	5.4/1.3	3.1/0.1	3.6/0.6
12	6.4/1.7	5.2/0.7	3.1/0.0	3.3/0.3
15	6.2/1.3	5.1/0.6	3.1/0.0	3.3/0.2
18	6.2/1.0	5.0/0.5	3.1/0.0	3.3/0.1
21	6.0/0.8	4.9/0.3	3.1/0.0	3.2/0.1
24	6.0/0.7	4.9/0.3	3.1/0.0	3.2/0.1
27	6.0/0.5	4.8/0.2	3.1/0.0	3.2/0.1
30	6.0/0.5	4.8/0.2	3.1/0.0	3.2/0.1

**Table 3:** Triangulation error (cm). The first column represents the number of training samples, and the first row gives the methods used for comparison. The refinement method uses the result of Orthogonal procrustes as initial guess, and then applies the proposed refinement method to improve the accuracy. The numbers are listed as “a/s” where a is the average over 100 experiments and s is the standard deviation.

	Rank-4	Orthogonal Procrustes	Refinement	Svoboda
03	N/A	4.8/0.8	4.5/0.7	N/A
04	5.5/1.6	4.7/0.7	3.5/0.4	N/A
05	5.1/0.9	4.5/0.6	3.4/0.2	N/A
06	5.2/1.1	4.6/0.7	3.4/0.1	N/A
07	5.2/1.0	4.7/0.7	3.3/0.1	N/A
08	5.0/0.8	4.5/0.6	3.3/0.0	4.2/1.6
09	5.2/0.9	4.6/0.7	3.3/0.1	3.8/0.6
12	5.3/0.9	4.5/0.4	3.3/0.0	3.6/0.3
15	5.3/0.7	4.5/0.4	3.3/0.0	3.6/0.2
18	5.3/0.6	4.5/0.3	3.3/0.0	3.5/0.1
21	5.2/0.5	4.5/0.2	3.3/0.0	3.5/0.1
24	5.2/0.4	4.5/0.2	3.3/0.0	3.5/0.1
27	5.3/0.4	4.5/0.2	3.3/0.0	3.5/0.1
30	5.3/0.3	4.5/0.2	3.3/0.0	3.4/0.1

**Table 4:** Reprojection error (pixel). The first column represents the number of training samples, and the first row gives the methods used for comparison. The refinement method uses the result of Orthogonal procrustes as initial guess, and then applies the proposed refinement method to improve the accuracy. The numbers are listed as “a/s” where a is the average over 100 experiments and s is the standard deviation.

	Rank-4	Orthogonal Procrustes	Refinement	Svoboda
03	N/A	4.5/1.7	3.3/1.6	N/A
04	6.1/5.5	3.9/1.3	1.2/0.8	N/A
05	4.8/2.5	3.6/1.3	0.7/0.4	N/A
06	4.6/2.3	3.6/1.4	0.7/0.3	N/A
07	4.3/1.8	3.6/1.3	0.6/0.2	N/A
08	4.0/1.6	3.3/1.3	0.5/0.2	0.8/1.1
09	4.3/1.7	3.4/1.1	0.5/0.2	0.5/0.2
12	4.4/1.5	3.2/0.6	0.5/0.1	0.4/0.1
15	4.1/1.1	3.0/0.5	0.5/0.1	0.4/0.1
18	4.1/0.8	3.0/0.4	0.5/0.1	0.4/0.1
21	3.9/0.6	2.8/0.2	0.5/0.1	0.4/0.1
24	3.9/0.5	2.8/0.2	0.4/0.1	0.3/0.0
27	3.9/0.4	2.8/0.2	0.4/0.1	0.3/0.0
30	3.8/0.3	2.7/0.1	0.4/0.0	0.3/0.0

We also performed some experiments on the calibration of a camera pair, *i.e.*, two cameras spaced far apart (not in stereo configuration). Methods such as the one of Svoboda *et al.* [5] cannot be applied in this case as they need a minimum of three cameras. Table 5 shows the result for camera 1 and camera 2. We calibrated using only nine training samples and evaluated based on the 40 test samples. The calibration accuracy after refinement is shown in Table 5, from which we can conclude that our method can still provide accurate calibration for camera networks with two cameras.

**Table 5:** Calibration accuracy of the network with only two cameras. The second row and the third row represent the mean and the standard deviation over all 40 test samples, respectively.

	$\delta u^{\mathrm{pr}}$ (pixel)	*δ***r**^*w*^ (cm)	$\delta u^{\mathrm{repr}}$(pixel)
mean	3.2	3.5	0.4
std	2.4	1.6	0.4

## 8. Conclusions

In this paper, we present a simple and robust method to compute the 3D positions of sphere centers w.r.t. the camera coordinate system by estimating only the center of the projected sphere and its area in an image. Then we propose to use orthogonal procrustes analysis to do pairwise calibration. Finally an optimization routine jointly refines the extrinsic parameters for all cameras.

Compared to other sphere based method which process the sphere images by tracing the contour of the elliptical projection of the sphere, our method provides simpler and more accurate estimation of sphere 3D positions w.r.t. local camera coordinate system. While the sphere positions are only used as intermediate results, a higher accuracy in these sphere positions still leads to a more accurate and stable estimation of extrinsic parameters.

Compared to the calibration method of Svoboda *et al.* [5], our method can provide more accurate estimation of extrinsic parameters with less training samples. We can also provide scale information assuming known sphere size. An additional benefit of our approach is that it can extrinsically calibrate two cameras at a time, whereas the method of Svoboda *et al.* requires at least three cameras. Moreover, the data capturing procedure is more simple and convenient for our method.

In the proposed algorithm and experiments, we assumed that all cameras in the networks share common volume, we chose one camera’s coordinate system as the world coordinate system and related all other cameras to it by pairwise calibration. Our algorithm can be very easily extended to the calibration of camera networks that do not share a common volume. In that case, only pairwise overlap is required. We will explore this in our future work.

## References

[1] Sturm P., Triggs B. A Factorization Based Algorithm for Multi-Image Projective Structure and Motion. Proceedings of European Conference on Computer Vision.

[2] Arun K., Huang T., Blostein S. (1987). Least-squares fitting of two 3-D point sets. IEEE Trans. Pattern. Anal. Mach. Intell..

[3] Bouguet J.Y. Camera Calibration Toolbox for Matlab. http://www.vision.caltech.edu/bouguetj/calib_doc/.

[4] Agrawal M., Davis L.S. Camera calibration using spheres: A semi-definite programming approach. Proceedings of IEEE International Conference on Computer Vision.

[5] Svoboda T., Martinec D., Pajdla T. (2005). A convenient multi-cameraself-calibration for virtual environments. PRESENCE Teleop. Virt. Environ..

[6] Hartley R., Zisserman A. (2003). Multiple View Geometry in Computer Vision.

[7] Pollefeys M., Koch R., Van Gool L. Self-calibration and metric reconstruction in spite of varying and unknown internal camera parameters. Proceedings of International Conference on Computer Vision.

[8] Triggs B., McLauchlan P., Hartley R., Fitzgibbon A., Triggs B., Zisserman A., Szeliski R. (2000). Bundle Adjustment-A Modern Synthesis. Vision Algorithms: Theory and Practice.

[9] Tsai R.Y. (1987). A versatile camera calibration technique for high-accuracy 3D machine vision metrology using off-the-shelf TV cameras and lenses. IEEE Trans. Robot. Autom..

[10] Faugeras O.D., Toscani G. The calibration problem for stereo. Proceedings of IEEE Conference on Computer Vision and Pattern Recognition.

[11] Hall E.L., Tio J., McPherson C., Sadjadi F. (1982). Measuring curved surfaces for robot vision. Computer.

[12] Weng J., Cohen P., Herniou M. (1992). Camera calibration with distortion models and accuracy evaluation. IEEE Trans. Pattern. Anal. Mach. Intell..

[13] Zhang Z. (2000). A flexible new technique for camera calibration. IEEE Trans. Pattern. Anal. Mach. Intell..

[14] Ueshiba T., Tomita F. Plane-based calibration algorithm for multi-camera systems via factorization of homography matrices. Proceedings of IEEE International Conference on Computer Vision.

[15] Aslan C.T., Bernardin K., Stiefelhagen R. Automatic calibration of camera networks based on local motion features. Proceedings of Workshop on Multi-camera and Multi-modal Sensor Fusion Algorithms and Applications.

[16] Sinha S.N., Pollefeys M., McMillan L. Camera network calibration from dynamic silhouettes. Proceedings of IEEE Conference on Computer Vision and Pattern Recognition.

[17] Penna M. (1991). Camera calibration: A quick and easy way to determine the scale factor. IEEE Trans. Pattern. Anal. Mach. Intell..

[18] Daucher N., Dhome M., LaprestÃ© J., Eklundh J.O. Camera calibration from spheres images. European Conference on Computer Vision.

[19] Teramoto H., Xu G. Camera calibration by a single image of balls: From conics to the absolute conic. Proceedings of The Fifth Asian Conference on Computer Vision.

[20] Ying X., Zha H. (2006). Geometric interpretations of the relation between the Image of the absolute conic and sphere images. IEEE Trans. Pattern. Anal. Mach. Intell..

[21] Ying X., Zha H. Linear approaches to camera calibration from sphere images or active intrinsic calibration using vanishing points. Proceedings of IEEE International Conference on Computer Vision.

[22] Zhang H., Wong K.Y., Zhang G. (2007). Camera calibration from images of spheres. IEEE Trans. Pattern. Anal. Mach. Intell..

[23] Eggert D.W., Lorusso A., Fisher R.B. (1997). Estimating 3-D rigid body transformations: A comparison of fourmajor algorithms. Mach. Vision Appl..

[24] Schönemann P. (1966). A generalized solution of the orthogonal procrustes problem. Psychometrika.

[25] Hartley R., Sturm P., Hlaváč V., Šára R. (1995). Triangulation. Computer Analysis of Images and Patterns.

